# Sequence Memory Constraints Give Rise to Language-Like Structure through Iterated Learning

**DOI:** 10.1371/journal.pone.0168532

**Published:** 2017-01-24

**Authors:** Hannah Cornish, Rick Dale, Simon Kirby, Morten H. Christiansen

**Affiliations:** 1 Department of Psychology, The University of Stirling, Stirling, United Kingdom; 2 Cognitive and Information Sciences, University of California—Merced, Merced, CA, United States of America; 3 School of Philosophy, Psychology and Language Science, The University of Edinburgh, Edinburgh, United Kingdom; 4 Department of Psychology, Cornell University, Ithaca, NY, United States of America; 5 The Interacting Minds Centre, Aarhus University, Aarhus, Denmark; Massachusetts Institute of Technology, UNITED STATES

## Abstract

Human language is composed of sequences of reusable elements. The origins of the sequential structure of language is a hotly debated topic in evolutionary linguistics. In this paper, we show that sets of sequences with language-like statistical properties can emerge from a process of cultural evolution under pressure from chunk-based memory constraints. We employ a novel experimental task that is non-linguistic and non-communicative in nature, in which participants are trained on and later asked to recall a set of sequences one-by-one. Recalled sequences from one participant become training data for the next participant. In this way, we simulate cultural evolution in the laboratory. Our results show a cumulative increase in structure, and by comparing this structure to data from existing linguistic corpora, we demonstrate a close parallel between the sets of sequences that emerge in our experiment and those seen in natural language.

## Introduction

A key ability of speakers and listeners is their capacity to “make infinite employment of finite means” ([1]: p. 91). To accomplish such open-ended productivity, humans exploit the “reusable parts” that make up language. It is therefore not surprising that the notion of structural reuse, in some form or other, plays a central role in many accounts of language, from linguistic grammars (e.g. [2]) and Bayesian approaches (e.g., [3]) to computational linguistics (e.g., [4]) and psycholinguistic modeling (e.g., [5]). Yet, it remains to be explained how languages come to be composed of reusable parts in the first place. Many factors are likely to have influenced the evolutionary emergence of reusable parts in language, including semantic information (e.g., [6]) and communicative pressures (e.g., [7]). In this paper, however, we focus on the need to arrange these parts with respect to one another [8], and the possible contribution of basic constraints on sequence memory as a driver of linguistic reuse. Specifically, we hypothesize that important aspects of the sequential structure of language, and its characteristic reusable parts, may derive from adaptations to the cognitive limitations of human learners and users.

### Sequence Memory and Language

Whether spoken or signed, language is serially produced and perceived at an incredibly fast pace.

Spoken syllables are produced at a rate of about 5–6 per second [9], while signed syllables have a duration of about a quarter of a second [10]. However, our memory for acoustic and visual information is very short-lived, disappearing in less than 100 milliseconds [11,12]. To make matters worse, even our memory for sequences of unelated spoken or signed linguistic items is limited to only four-to-seven items [13–15]. Thus, during normal linguistic interaction, we are faced with an immense challenge by the combined effects of rapid input, short-lived sensory memory, and severely limited sequence memory. As a consequence of this *Now-or-Never bottleneck* [16], new material will constantly overwrite and interfere with previous material unless it is processed immediately.

The basic memory process of chunking [14] provides a possible way to overcome the constraints imposed by the Now-or-Never bottleneck. Through linguistic exposure, language users learn to do *Chunk-and-Pass* processing [16]: compress and recode language input as rapidly as possible into increasingly more abstract levels of linguistic representation, from sound-based units to words (or word combinations) to discourse-level representations. This passing up of chunks allows for increasingly longer retention of linguistic information at higher levels of linguistic abstraction, in line with recent neuroimaging data (e.g., [17,18]). Thus, the reuse of chunks across the different levels of linguistic representations provide a possible way in which language might achieve its open-ended productivity. Consistent with this perspective, there has been a growing body of work demonstrating a key role for multiword chunks as building blocks for both the acquisition (e.g., [19–21]) and processing (e.g., [22–24]) of language. Here, we employ iterated learning to further investigate whether chunking, as a basic mechanism of memory, might contribute to the emergence of language-like distributional structure. In doing so, we suggest that language evolves culturally in such a way that its structure provides a solution to the Now-or-Never bottleneck.

### Cultural Evolution in the Lab

Recent years have seen the emergence of various experimental techniques for lab-based explorations of questions related to the cultural evolution of language. Many of these studies have sought an understanding of the origins of language as a product of cognitive and cultural processes (see [25] for a review). These studies attempt to link observed features of language, such as compositionality [26] or duality of patterning [27], to such processes by demonstrating how they can emerge as a consequence of language learning and interactive use by participants over time in controlled laboratory settings. Other factors like population structure (e.g., [28]) and the structure of the meanings in the world (e.g. [29]) have also been shown to have a major effect on the kinds of structure that emerge.

Most of these studies leave open the question of whether any aspects of linguistic structure can emerge independently of the structure in the meanings being conveyed. Furthermore, these factors have tended to be studied using tasks that are, in their instructions, either overtly linguistic (participants are told they are using a language, and given data upon which to make linguistic observations) or communicative (participants are encouraged to create a system to exchange information). This gives rise to a potential issue affecting all of these studies, namely, the degree to which they can be explained as a result of the adult human participants already possessing a language. A common argument that leads some researchers to question the viability of carrying out experiments investigating the origins of language (e.g., [30]) is that the key result of structural emergence is already built into the research paradigm by virtue of there being pre-existing biases from social or linguistic cues.

Researchers have attempted to address this criticism in various ways. One suggestion is that these experiments could be run on pre-linguistic children and non-humans [31]. Although there are strong methodological challenges associated with these approaches, work has begun in this area, most notably with iterated learning studies on zebra finches [32] and baboons [33]. Another approach is to move the task away from standard communication channels in order to reduce any interference from underlying language competences (e.g. [34]). Though this is a good idea in principle, a problem is that the underlying tasks are still communicative in nature, and are therefore likely to recruit from known systems of communication regardless of a change in modality or medium. The current study was therefore designed specifically to be non-communicative in nature and not to rely on existing language skills.

### The Current Study

Our study was explicitly designed as a memory experiment involving the exposure to nonsense sequences of letters, in the absence of any communicative task demands or need for language skills (except to understand the instructions). We wanted to explore whether the basic memory process of chunking would lead to reuse of parts as a result of cultural transmission without a communicative or a linguistic task being required. Will structure emerge when the only pressure is coming from domain-independent sequence learning constraints? In our setup, there are no meanings or referents to convey, no interactive elements between learners, nor is communication implicit in the instructions. Indeed, the instructions explicitly framed the study as a memory task where the only goal was to recall a set of sequences seen during a training phase. The recalled sequences are then used as training items for the next participant, and the process is repeated for 10 “generations”, creating a linear diffusion chain of learners.

Our primary hypotheses are that (a) sequences will become more learnable over time, (b) their distributional structure will increase, and importantly, (c) they will take on structural properties that have language-like features, such as the reuse of parts. The upshot, which we revisit in the Discussion, is that the basic chunk-based constraints on sequence memory, amplified culturally in the laboratory, induces the emergence of language-like structure—without any linguistic or communicative constraints. Language may, too, be shaped by these constraints. Linguistic structures must be kept distinct to convey distinct meaning, yet must accommodate a limited memory system. The conclusion is that these basic cognitive processes may be partly responsible for the structure of human language [16,35].

## Method

### Participants

This experiment was approved by the Linguistics and English Language Ethics Committee at the University of Edinburgh, and written consent was obtained from all participants before taking part. For all iterated learning experiments a decision has to be made in advance as to how many groups (or “chains”) to run, and how many participants (or “generations”) each chain will contain. We followed established practice by running for ten generations (c.f. [26,36]), and opted for eight chains in total. Eighty adult University of Edinburgh students (age: *M =* 21.72*; SD =* 4.08) each received £2 for their participation, and were randomly allocated to one of the eight chains. As described below, a chain involved 10 participants, run separately and sequentially in the task, where one participant’s behavior served as input (or stimuli) for the subsequent participant.

### Materials

Participants were told that they would be administered a memory task, involving a series of to-be-recalled consonant letter strings. To provide the training items for the first participants in each of the eight chains, eight initial string sets were generated. A string set contained fifteen strings in total, with five strings of length three, four and five respectively. The construction of these initial string sets was tightly constrained to ensure there were no sequential patterns to bias learners toward a particular structure from the outset. Each string set contained exactly six consonants, each appearing ten times, yielding sixty letters in total distributed across the fifteen strings. The identity of the letters differed between sets, having been randomly drawn from the full set of 20 (capitalized) consonant characters available on an English keyboard. Crucially, throughout the string set, bigram and trigram frequencies were kept as near uniform as possible. In practice, this meant that no more than three repetitions of a single bigram, and two repetitions of a single trigram, were permitted. This results in string sets which are both randomly constructed, yet also unstructured. We designed 8 initial string sets for each chain of 10 participants (see Table 1).

**Table 1:** The initial string sets for the first participant in each of 8 chains.

Chain	String set
**1**	CMC, SFL, PCS, LFF, FSM, MSMF, CLMP, PPSL, FLCM, SCPC, CSPLL, LFPSS, PFMLM, MLCFP, SPMCF
**2**	VSB, SGT, GTV, BVT, TBZ, VBSS, GZTB, STGS, TZBT, ZVTG, BZTSV, VBGSZ, GVVZG, SSGBB, ZGZVZ
**3**	SLW, LXS, CWC, WSX, XKK, LSWK, CCCX, KXKL, SXLC, WKXL, KSKCW, SWCLX, WLSCS, LWXSC, XWLKW
**4**	JNB, FJQ, QFP, PPN, NJF, JPFQ, QBNF, FQBP, BFFB, NJBN, JPQNP, BQPBB, PFJNQ, NQNBJ, FPJQJ
**5**	XLJ, NXQ, LQP, PNN, JPL, QJNX, PQLQ, XPJL, LNQN, NJXJ, JNPXP, LXJQJ, PLXNQ, QQLPN, XLJPX
**6**	PCH, NVP, VNC, HPV, TCN, NPTN, TVTP, HCNT, CTHV, PHHC, NHTCT, TVHPH, HVPCV, CPNNC, VCVNP
**7**	RLB, VBF, LFR, GGV, BRG, RBGL, LFBV, VLGG, GFLL, FGLB, GBVRF, BLVFF, LVRRB, RVFBR, FVGRV
**8**	SRS, ZPR, MRL, RZM, LMZ, RRZR, LPMP, PLRM, ZSMM, SLSP, PZPSS, MLZRL, RPMPZ, SZLLZ, LSMSP

### Procedure

The 80 participants in this task were organized into 8 chains. In a chain, the first participant received one of the initial string sets in Table 1. The memory test result for this participant served as the stimuli for the second participant; this second participant’s final test result served as stimuli for the third; and so on, up to the tenth participant. Eight of these iterated learning chains were run to investigate the effect of sequence learning constraints on the learnability and structure of the sets of strings as they changed over time.

Unlike typical iterated learning experiments (e.g., [26,37]), the strings to be acquired by learners had no associated semantics, and were not used in a linguistic or communicative context. Instead, participants were informed that they were taking part in a memory experiment. At no point were the strings referred to as a ‘language’, nor were learners aware that their output was to be passed on to a subsequent participant.

A chain consisted of ten “generations” of learners. At each generation, a participant first underwent an implicit learning regime (“echo training”) to acquire a finite set of strings, before being prompted to reproduce the items they had seen in a final test. The output of this final test was then used as training input to the next learner taking part in the experiment, thus adding a generation to the chain. In total, echo training and testing lasted no more than 15 minutes.

During echo training, participants were exposed to six blocks of the fifteen strings, presented in random order. Each string appeared onscreen for exactly 1000ms. After a 3000ms delay, participants were prompted to type in the string using the keyboard. If participants attempted to echo the string before the end of the delay, the keyboard would fail to register the input and a warning beep would sound. No feedback was provided on the correctness of the entered string.

After training, participants were given a surprise test. They were told how many strings they had seen during training, and were then asked to recall each one as best they could. Participants entered the strings one-by-one and were given no feedback on the accuracy of a recalled string. The screen was cleared between each recall attempt. The only information provided was a counter indicating the number of strings that they still needed to produce. The sole requirement for this final test was that each produced string be unique. If a string was typed in more than once, an error message appeared and participants were instructed to try again. The 15 unique strings retrieved at the end of recall were transmitted to the next participant for learning in all cases except for the first learner, who received an initial string set that was randomly constructed (Table 1).

To avoid potential biases that might affect the learning process, we implemented a re-mapping procedure to remove any surface structure effects. For example, acronyms might be introduced into the strings by participants, or the physical distribution of letters on the keyboard could lead to the emergence of certain typing patterns. To counteract these biases, the string sets were re-mapped to new consonant characters at the end of each individual test session (e.g., each instance of *X* might be replaced by *N*, *and so on*). The output was then visually inspected by a native English speaker before being transmitted to the next generation. If an acronym was found, the re-mapping process was repeated until an acronym-free assignment of characters had been found. This process results in the removal of confounding surface regularities, whilst preserving the underlying structure of the string sets.

## Results

To test our hypotheses, we conducted several different analyses, looking at increases in learnability, the emergence of distributional structure, and comparing structural reuse patterns with those found in child-directed speech as well as in other human-generated sequences. In each case, we leveraged a different kind of structural analysis which had explicit predictions rendered in advance of the test.

### Learnability Increases

In order to determine whether string sets are being acquired more faithfully over time, we computed the overall accuracy of the items recalled across generations in terms of the normalized edit distance [38] between strings in generation *n* and *n* + 1. Following a standard approach used in artificial grammar learning to compare the similarity of test items to training items [39], we determined for each recalled test string (at generation *n* + 1) which of the training items (from generation n) *that* it was closest to. For example, if a recalled item QZM has QZV as its closest training item then it would be assigned an error score of 1. This score reflects the minimum number of edits (i.e., insertions, deletions or substitutions) required to change a test item into the closest training item. The global error score for a given generation was computed as the mean edit distance across all the recalled items. The lower the mean error score is, the more similar the items in generation *n* + 1 are to those in generation n. More accurate recall thus results in lower error scores.

**Fig 1:**
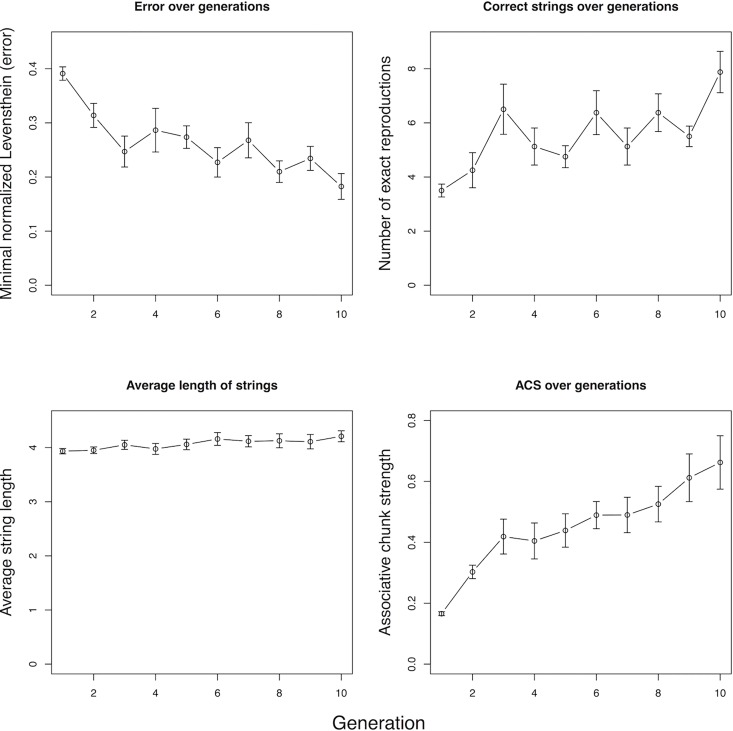
Increase in learnability and distributional structure across generations of learners. Global error decreased across time (top-left). Participants become better at reproducing the string sets (top-right). String sets do not diminish in length across time (bottom-left). Structure increases over generations, as indicated by the mean of Associative Chunk Strength (ACS) of string sets (bottom-right). In all cases, the graphs plot means across all eight chains, with error bars reflecting standard error of the mean.

Fig 1 (top-left) shows a graph of how global error changes over time, averaged across the eight chains. A paired samples *t*-test comparing global error scores from the initial generations with those of the final generations, revealed that there is a significant decrease across generations: string sets were generally recalled more accurately at the end (*M* = 0.18, *SD* = 0.08) of chains compared to the beginning (*M* = 0.39, *SD* = 0.04); *t*(7) = 5.82, *p* < .001. The boost in overall accuracy translates into a significant increase in the number of correctly recalled items, from a mean of 3.5 (*SD* = .76) at generation 1 to 7.9 (*SD* = 2.42) at generation 10; *t*(7) = 4.73, *p* = .002 (Fig 1, top-right). Importantly, the improved learnability did not come at the cost of a collapse of the string sets into very short sequences (Fig 1, bottom-left). There was no difference in the mean length of the strings when comparing initial (*M* = 3.93, *SD* = .16) and final generations (*M* = 4.21, *SD* = .32); *t*(7) = -2.27, *p* = .06. Indeed, there is a slight trend for strings to become longer. We also tested trends across generations using linear mixed effects models with maximized random-effects structures [40]. All trends are robust (*p* < .001) with the exception of string size, which shows a statistically marginal tendency to *increase* across generations (*p* = .08). The contrast among measures shown in Fig 1 is striking. If anything, strings are increasing in length, yet participants are recalling them more effectively. Our next analyses answer the question how such an encoding could become more efficient despite the increasing length.

### Distributional Structure Increases

Our learnability analyses indicated that the string sets became easier to learn across generations. To determine whether this increase in learnability was driven by the emergence of distributional structure, as we had hypothesized, we adopted a metric frequently used in artificial grammar learning studies: *Associative Chunk Strength* (ACS) [41]. ACS provides a simple measure of how distributionally similar a test item is in terms of its component chunks to a set of training items. For a given test sequence consisting of *x* bigrams (pairs of consecutive elements) and *x—*1 trigrams (triples of consecutive elements), ACS is calculated as the relative frequency with which those chunks occur in the training items. For example, ACS for the recalled item ZVX is calculated as the sum of the frequencies of the fragments ZV, VX and ZVX divided by 3. In our particular case, the training items are simply the strings in generation *n—*1, as we are comparing the amount of change in the distribution of chunks between successive generations. We calculate the amount of reuse in chunks over the entire string set, averaging the ACS across each test item (i.e., each string in generation *n*) in the set. This provides us with a global ACS measure that gives us an indication of how much repetition there is of sub-elements in our string sets, and consequently, how structured each system is.

Fig 1 (bottom-right) indicates that the amount of reuse of chunks (structure) increases considerably over time. We also find a significant difference between the first and last generations, in that generation 10 (*M* = 0.66, *SD* = .28) shows more chunk reuse than generation 1 (*M* = 0.17, *SD* = .02), *t*(7) = 5.0, *p* < .005. A similar linear mixed effects model described in the last section confirms a trend to increase over generations (*p* < .0001). In other words, relative to the previous generation’s chunks, the next generation tends to reuse these chunks successfully, and more so as generations proceed. The participants are developing re-usable units incrementally.

### The Emergence of Language-Like Structure

The analyses performed so far support our hypotheses that distributional structure which facilitates learning emerges as a result of cultural transmission over time, but we still need to determine whether that structure is at all language-like. To do this we performed a network analysis on the experimental data and compared it to the same analysis on a corpus of natural language. The CHILDES corpus contains a collection of transcripts of both child language and child-directed speech [42]. We compare the networks derived from the experimental results to one based on the English child-directed speech portion of CHILDES to determine if there are some common structural properties that underlie both (please see https://github.com/racdale/cornish-strings to view data files, models, and methodological information used to perform this analysis in more detail).

There has been a recent rise in interest in looking at natural languages using methods from network theory (for a review, see [43]). A general motivation for using these techniques is that they permit quantification at a system level, by revealing the interrelationship among components of a language. For example, [44] explored processing implications of a lexicon characterized as a network of words connected by shared phonological properties, and [45] explored properties of sentences expressed as a network of words connected by sequencing. In general, network methods permit both visualization and quantification of the structural properties of language at various levels. We conducted the same analyses of the experimental data and the CHILDES corpus: If structure reuse increases, then network properties should evolve across generations. As we detail below, if we consider two strings to be “connected” on a graph based on whether they share a subsequence (such as a bigram), we ought to find that gradual reuse across chains leads to more densely connected networks of strings. To compare this to a baseline, we can shuffle these strings internally, thus removing the sequential structure. We predicted that the experimental data networks should come to resemble the CHILDES network.

#### Experimental networks

**Fig 2:**
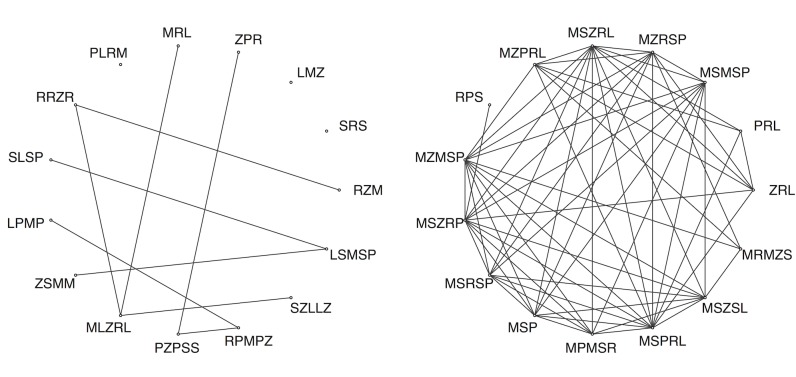
Generations 0 (left) and 10 (right) of chain 8. These network diagrams link strings that share at least one bigram sequence. Although the string sets start out containing relatively few edges (links), by the end of the chain the strings have become quite densely connected to one another.

Because each generation consists of only 15 strings, we assessed emerging *shared* structure in networks by assessing the extent of interconnection among string sets across generations of learners. We used a very simple definition of connectivity among strings of a generation: Two strings are connected to each other if they share at least one letter-bigram chunk. An example network is shown in Fig 2. If participants are gradually structuring the strings so that they are more memorable (yet distinct), from generation to generation, strings may come to exploit sequential patterns. This hypothesis is indeed suggested by the ACS analysis above, but in the case of the emerging networks across a chain, the hypothesis would be confirmed by the strings becoming more and more interconnected by shared chunks.

#### CHILDES natural language networks

For the purpose of our natural language analyses, we extracted the English child-directed speech from the CHILDES corpus. Adults normally use a considerably larger number of words when speaking to children than the few letter types used in our experiment. To reduce the number of element types to be more in line with the experiment, we therefore replaced individual words in the child-directed utterances with their respective parts-of-speech (POS) tags, drawn from a set of fifteen: noun, verb, adjective, adverb, determiner, preposition, negation, conjunction, pronoun, relativizer, quantifier, onomatopoeia, interjection, infinitival, neologism. The resulting strings represent the manner in which parts of speech are encoding messages sequentially. In other words, just as our experimental string sets are composed of a small number of letter types, natural language sentences can be described in terms of a small number of parts of speech.

We built the natural-language network in a similar way to what we described above: Any POS string (e.g., *noun-verb-preposition-noun*) is connected to another if they share a bigram (e.g., *noun-verb*). We chose the 10,000 most frequent sequences (77% of the total CHILDES strings), and extracted those with length similar to our experimental strings: 3 to 6 (*N* = 6,266). In terms of the overall corpus of *all* POS strings (*N* = 237,575, with 1,243,472 token frequency), these 6,266 strings represent approximately 41.5% of all utterances by frequency (515,874 token frequency). We constructed a single network based on this large set of strings.

#### Statistical baseline networks

For both experimental and natural-language networks, we also constructed a statistical baseline by taking the same string sets but shuffling the elements within each string before building the network. This removes the sequential structure of a given string and should disrupt the interconnectedness of the resulting network. We did this once for each network, serving as one shuffled comparison.

#### Comparison of shared structure

A simple consequence of creating networks by linking strings that share bigrams is that, as strings get longer, they are more likely to have connections to other strings. This would be the case in both the experimental networks, and natural-language networks. In fact, we predicted that this connectivity, as a function of size, should be similar if our experimental data involve chunk reuse in a manner similar to language. In other words, proportional increase in string size should, if structural reuse is taking place, show similar increases in connectivity (compared to baseline).

**Fig 3:**
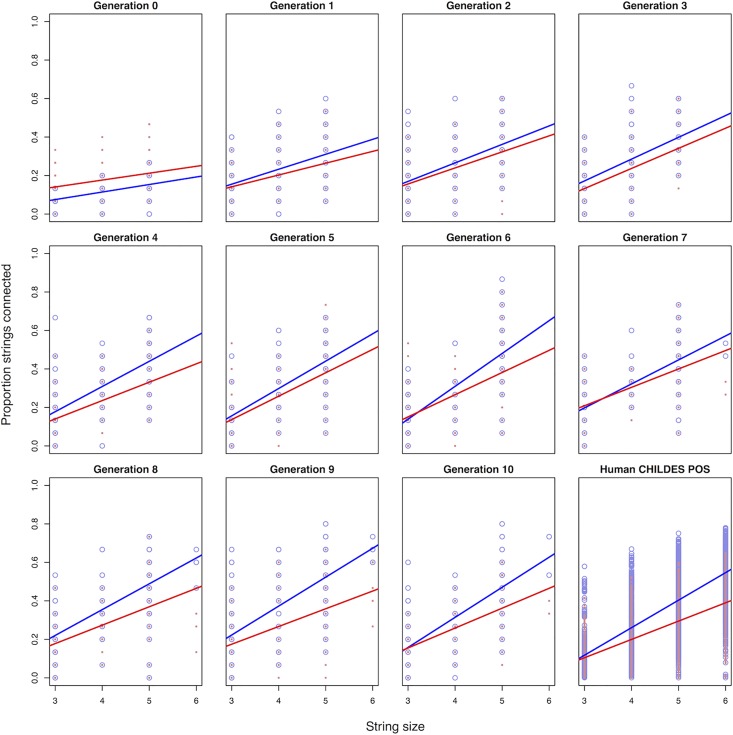
From top-left to bottom-right demonstration the emergence of interconnected structure of strings by bigrams. By comparison to natural language part-of-speech (POS) ordering from CHILDES (bottom-right panel), the relationship between string size and shared bigrams resemble each other closely. Blue circles are items from the original data; red dots reflect string-internal shuffled items. Lines are linear fits with corresponding color designations.

For each set of networks, both experimental and natural-language (and their baselines), we extracted (1) string length, and (2) the *proportion* of other strings in the set to which a given string is connected. The relationship between these variables is shown in Fig 3, with blue lines indicating experimental/CHILDES data and the red lines the corresponding shuffled baselines. For the natural-language (CHILDES) network, the original data (unshuffled) have overall greater connectivity than the shuffled data by (on average) 10%, *t* = 47.6, *p* < .0001, and the interaction in Fig 3 (bottom right) is significant, *t* = 20.7, *p* < .0001. Importantly, these effects are still present when just focusing on strings of length 3 and 4 alone: It is not driven exclusively by the longer string sequences (*p*'s < .0001). This reveals that the observed CHILDES sequences are sharing bigram chunks, giving way to patterns of reuse relative to a shuffled baseline.

We did this same analysis across our generations of the experiment, shown in Fig 3. In the first panel, Generation 0, the shuffled strings (red) are in fact significantly greater in their overall connectivity, *t* = 6.3, *p* < .0001. This gradually changes, and by the final three generations (8, 9, 10) the original data are more greatly connected as a function of string length, *t*'s > 2.5, *p*'s < .005. Strikingly, the connectivity of the late-generation experimental networks is greater than the shuffled ones, on average, by a similar percentage to the natural-language network (7–11%). By the final generation (10), the interaction term reaches statistical significance. Though a weaker result, it suggests that connectivity scales with length differently relative to the shuffled baseline, even in these experimental data, *t* = 2.8, *p* < .01. This would be predicted by reuse of chunks: As strings increase in length, there should be an increased chance of sharing structure with other strings. The interaction term reveals that this scaling occurs in the experimental data.

We can now compare the human part-of-speech data to the experimental data directly, because they can be compared on the same scale (proportion of connectivity). In the final three generations (8,9,10), the CHILDES data does statistically differ from the experimental data in extent of connectivity. In particular, the experimental data are more connected, by about 9% (*p* < .0001). This is likely because the POS CHILDES data involve more categories (parts of speech), and thus more bigram types, and lower probability of drawing edges between sequences. Importantly, the interaction term in this analysis is not significant (*p* = .72), so we cannot infer a slope difference between CHILDES and the experimental data in later generations. However, the CHILDES data do differ from the first three experimental generations considered together (1,2,3). The CHILDES data show considerably more connectivity, and the interaction term is significant (*p* < .0001), suggesting that natural-language connectivity scales more robustly with length than the first few generations of the experiment, but more similarly to the final three generations.

#### Comparisons to other types of sequence structure

The global nature of the comparisons between the experimental and CHILDES networks raises a concern that the scaling of chunk reuse with length might be a general property of human-produced sequences. That is, the observed similarities might be a trivial consequence of strings being generated from a limited set of elements rather than structural reuse due to chunk-based memory processes common to both language and sequence learning, as we have suggested. To address this concern, we repeated our network analyses with three additional types of human-generated sequences: word frequencies, passwords, and random numbers (see further details in S1 Text).

Word frequency is an important factor in language processing. Using a subset of 5,000 words from Google Ngram (from [46]), we treated the frequency of words as digit-sequences. For example, the word “memory” had a frequency of 215,686 in 2008, which was used as a string of length 6 (i.e., “215686”), and connected to frequency counts of other words, given its five component digit bigrams (i.e., 21, 15, 56, 68, 86). In the same year, the word “string” had a frequency of 83,915 (bigrams: 83, 39, 91, 15), which shares the digit bigram 15 with “215686” and the two number sequences were therefore connected in the network. The digit sequences used to create the resulting network were not directly generated by humans but, rather, are an indirect reflection of the overall frequency of sequential usage patterns across many people. We would therefore not expect these sequences to show the kind of reuse we observed for the final-generation experimental networks and the CHILDES networks. This is a relatively weak baseline, because it would be surprising and unintuitive for such strings to exhibit distributions akin to structural reuse. Nevertheless, this initial baseline would demonstrate that not all natural distributions of strings show connectivity-by-length scaling.

**Fig 4:**
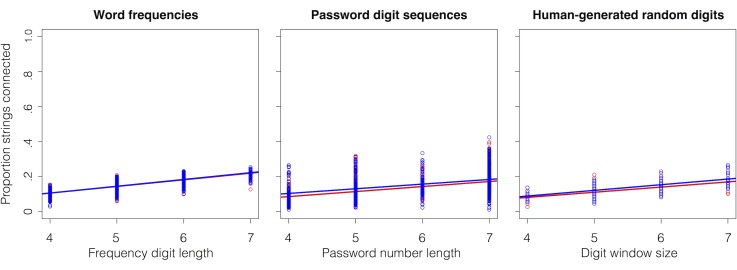
Network connectivity analyses of three different types of sequences: word usage frequencies treated as sequences (left panel), digit sequences gleaned from passwords (center panel), and human-generated sequences of random digits (right panel). Blue circles are items from the original data; red dots reflect sequence-internal shuffled items. Lines are linear fits with corresponding color designations. Only the sequential generation of random digits reveals the same pattern as observed in Fig 3 for late-generation and CHILDES networks.

As shown by the left-most panel in Fig 4, there is no evidence of robust scaling of chunk reuse in the frequency network as evidence by the lack of connectivity difference between the observed and shuffled conditions. Using the same regression approach as for the experimental and CHILDES data, we find that its interaction term is not significant (*p* = .14; see Supporting Information for more detail).

It is possible, though, that any sequence directly generated by humans will show the kind of scaling of reuse reflected by Fig 3. To investigate this possibility, we randomly selected 5,000 passwords, and extracted the numeric sequences contained within them, resulting in approximately 1,000 digit sequences. Such passwords are individually generated, typically to be memorable for the specific person using it. We created a network using password digit sequences, connecting two passwords if they contained similar digit bigrams (similar to the frequency networks). For example, “1492” and “123456” are common passwords with no overlap in digit bigrams. The resulting password network was then analyzed for chunk reuse as before. As can be seen from the center panel in Fig 4, there is a very slight advantage of the observed password sequences over the shuffled controls (*p* = .02). However, this advantage does not increase with length, as previously observed for the experimental and CHILDES networks (*p* = .34).

Remembering self-generated password typically involves recalling a single string. However, perhaps the patterns of chunk reuse seen in Fig 3 might be a simple consequence having to produce multiple strings, independent of whether they contain any notable structure? To test this, we obtained data from a random number generation task [47]. In this study, participants randomly generated numbers between 1 to 10 across 100 consecutive trials. To obtain strings for our analysis, we combined data from 5 of these participants. This produced one long string of digits 1–10. We then resampled segments from this long string of digits by extracting windows of length 3 to 6, to match our original experiment. This produced a set of strings, approximately 100 such sequences of length 3 to 6, that can then be subjected to our network analysis. Fig 4, right panel, shows that sequential generation of random numbers does not give rise to the kind of scaling pattern we found for the experimental and CHILDES networks. Although there is an increase in connectivity with length, the key interaction between the shuffled and random number networks is not observed (*p* = .5).

We ran this analysis on many iterations of the random number sequence, and the same result obtains. We would not argue, of course, that this means that the random number generation of the participants is truly random; other studies have shown that non-random patterns can infiltrate these number generation tasks, depending on how the task is set up, and how performance is measured (see [48–50]). However, our network analysis may be suitable to determine reuse of structure, in a language-like manner, rather than simply non-random structure, in a more generic information-theoretic sense. These concepts are distinct, and though it is outside the scope of the present analysis, it may be interesting to explore them in follow-up analysis.

Together, these three network analyses suggest that the similarities in the scaling of chunk reuse between the late-generation experimental networks and the CHILDES network is not a general property of sequences arising from human behavior. All three sequence networks—based on frequency, passwords, and random numbers—do not show the same reuse scaling as seen in Fig 3, even though they arise directly or indirectly from human sequential behavior. It appears that only chunk-based memory processes related to the learning and processing of multiple sequences involving some sort of structural relationship to one another result in the kind of structural reuse that we have proposed may common to both language and sequence learning.

### Example String Sets

**Fig 5:**
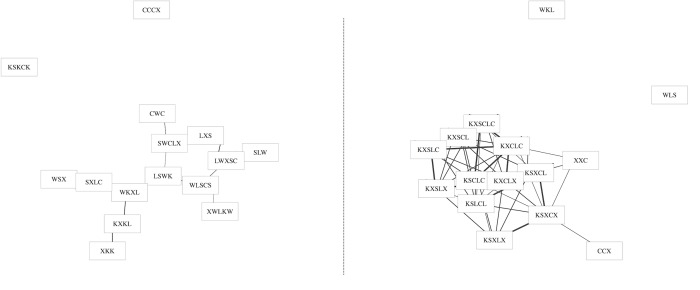
Examples of string sets found in the experiment. The initial string set for chain 3 at generation zero (left panel) is lacking in structure, with many singletons. Connections are present when there are shared bigrams. The same string set from chain 3 transformed by the participant chain after ten generations (right panel). Using an automated Kamada-Kawai force-directed method, strings can now be grouped together based on structural similarities. The width of the edges on the network reflect string-edit distance—structural similarity. In general, we find similarity among clusters to increase and take on some apparent systematic structuring.

We can see the process of chunk-based reuse occurring more clearly by examining the string sets qualitatively. Fig 5 shows an example of an initial random string set (left), and what that same string set evolved into after ten generations (right). We organized strings by combining the string-edit distance measure described above with the bigram connectivity rule used in the previous section. This allows us to organize string sets automatically, using a force-directed Kamada-Kawai algorithm [51] on our network, and qualitatively interpret what patterns appear present. Nodes that appear close together in Fig 5 reflect strings that they are part of a motif or “clique” using similar encoding strategies. The width of the line connecting these strings is proportional to the inverse string edit distance.

This automated technique allows visualization by grouping strings sharing similar features; it is important to remember that many other interpretations are possible and this is not necessarily how participants themselves would categorize items. With this caveat in mind, we can see that by the end of the experiment it is possible to discern certain patterns of organization. Some strings are “singletons,” and are unique with respect to their whole group. However, such singletons are relatively rare. Instead, we see much clustering and shared structure. For example, in Fig 5 (right), we see that a cluster of strings has the same initial bigram. The initial bigram *KX-* is used on the left side of the main cluster, and near the bottom-right side we see strings using *KS-*. The pattern of usage appears to involve some initial forms of transformation. Some of the strings on the right can be converted to those on the left by inverting the order of “X” and “S.”

## Discussion

In the experimental task, participants were not cued to think of the string sets as having communicative or linguistic relevance. Inspired by a long tradition in memory research, from Ebbinghaus onward, we utilized the well-known letter-string recall task. Participants were trained for several blocks on 15 strings, and then asked to reproduce them; the recalled strings served as input to the next participants across 8 chains of 10 subjects. We find that this classic memory recall context nonetheless induces the kind of “structural reuse” seen in natural language. Across generations, strings come to rely increasingly on a decreasing number of chunks, which significantly improves memory performance. They come to form a kind of structured system, involving the reuse of chunks in systematic ways that exceeds what one would expect from random strings. In addition, it seems that the emerging structure among the string sets has some properties in common with natural language. Analysis of a large-scale CHILDES data set shows that the shared structure among strings scales similarly with string length when we compare child-directed speech to the experimental string sets. Further comparisons with other types of human-generated sequences further underscore that it is chunk-based memory constraints associated with the repeated generation of sequences that result in such structural reuse. This gives support to the proposal that iterated learning leads to structure in language that helps alleviate the challenge posed by the Now-or-Never bottleneck.

The comparison of our string sets to the data contained in the CHILDES corpus has at least two limitations. The first has to do with the relative lengths of string and size of ‘language’ represented by our string sets. The second is a question of what our string sets might plausibly represent in language, given the lengths we have gone to in ensuring that our data be as non-linguistic as possible. We do not make any strong claims about the second issue. Though there are units of linguistic structure that carry no semantic information at all (phones), our character strings were not designed to resemble this aspect of language. However, it seems plausible that the process of constraining structural reuse via cognitive processes such as chunking likely holds across a range of levels in linguistic organization [16,36]. What we have demonstrated here is that echoes of this process can hold even in a very simple experimental design, without any overt linguistic framing or semantic constraints present.

From the admittedly limited standpoint of parsimony, the results suggest that the constraints on cognitive processing *alone* could offer an elegant account of how sequence structure emerges [52]. In particular, the constraints on encoding and recalling sequence elements from memory may serve as a kind of “filter” that biases the transmission of structures from participant to participant [53]. This biasing leads to a set of strings that are assembled from reused parts, and still permit distinctiveness across the whole set of strings. This distinctiveness is forced in our experiment (participants had to produce 15 unique strings); in the communicative context, a lack of distinctiveness would be subject to more natural contingencies, such as in referential expressions, to avoid potential ambiguities.

These principles of memory encoding and recall may offer an explanation of the balance between (a) emerging sub-structures that permit efficient use of memory while also (b) preserving distinctiveness among the entities to be learned and reproduced. This reflects a kind of intermediate strategy between maximal *encoding efficiency* (15 almost identical strings), and *maximal distinctiveness* (each string highly different from the rest). Participant chains find a balance between these forces. Importantly, though, we do not wish to argue that this way of seeing linguistic structure as the result of a trade-off between competing forces of efficiency and distinctiveness is entirely novel. The idea that language adapts to meet functional challenges has been developed in various ways in many areas (among many others: [54–58]). Our results provide experimental evidence regarding the way in which simple memory constraints may give rise to distributional sequence structure.

We do not deny the importance of semantics and social coordination in language use. In natural contexts, communication serves as an additional and equally important constraint operating alongside other perceptual and cognitive constraints. The need to have another person produce and understand a set of sequential structures requires maintaining a certain amount of distinctiveness in the system. So, the generational transmission of language filtered through cognition likely operates alongside a process of social coordination that biases structural encoding and distinctiveness as well (e.g., [59]). We would not advocate that cognitive constraints trump sociocultural coordination; to us they seem part and parcel of the same system (see, e.g. [60], for discussion). Language may be conceived as a communication system shaped by selective pressures from multiple cues and constraints. Languages take on various forms, at various levels, that adapt to contexts in which a language is used [35].

These same caveats could be expressed for linguistic meaning, omitted deliberately from our design. It is likely that this balance between efficiency and distinctiveness is constrained by the meanings to be expressed. It has long been known that competition among similar forms may lead to distinctive encodings, especially at the phonological level. For example, in conditions of potential ambiguity at the lexical level, language users amplify subtle phonological distinctions in order to render more clear the distinction among lexical items [61]. Simulations of this process show that this may lead to an iterative process of change as well, thus similarly explaining phonological systems as having some emergent structure [62].

More generally, our results relate directly to another mechanism proposed in human memory research that has a long history. Chunking continues to be regarded as a fundamental process for rendering large amounts of information more easily memorable by restructuring or reusing components on which that information is based. For example, in Miller’s classic study [14], he observed how “recoding” can permit a person to recall a sequence as long as 40 binary digits. The strategy, of course, is to accommodate limits in memory by engaging in reuse of parts, and then manipulating those parts permits a more efficient encoding of, on its surface, very lengthy or detailed material. Chunking has a long history since this classic work, and continues to figure prominently in our understanding of human learning and memory [63], including in the acquisition of language [16,64,65].

## Conclusion

We began our paper with a description of language as one which makes infinite use of finite means, “reusing” structures in systematic ways that permit generalization and application in many contexts. In our experiment, we demonstrate that some aspects of structural reuse may emerge under cognitive constraints, driven only by the demands in a basic memory task, devoid of communicative or semantic dimensions. Of course, our results cannot yet approximate the “infinite employment” described famously by Humboldt. But our findings do offer an important clue to how “finite means” may come about, and the way they work to cognitively support our productive linguistic abilities.

## Supporting Information

S1 Text Supporting Material: String Length-Connectivity Scaling. (DOCX)

## References

[1] von Humboldt W. On language: On the diversity of human language construction and its influence on the metal development of the human species Cambridge: Cambridge University Press; 1999/1836.

[2] Culicover P. Grammar and complexity: Language at the interface of competence and performance. Oxford: Oxford University press, 2013.

[3] O'Donnell TJ. Productivity and reuse in language: A theory of linguistic computation and storage. Cambridge, MA: MIT Press, 2015.

[4] Lignos C, Gorman, K. Revisiting frequency and storage in morphological processing. Proceedings of the 48th Annual Meeting of the Chicago Linguistic Society, 447–461, 2014.

[5] Chater N, McCauley SM, Christiansen MH. Language as skill: Intertwining comprehension and production. J Mem Lang. 2016; 89: 244–254.

[6] Hills TT, Adelman JS. Recent evolution of learnability in American English from 1800 to 2000. Cognition. 2015;143: 87–92. 10.1016/j.cognition.2015.06.009 26117487

[7] Wray A., Grace GW. The consequences of talking to strangers: Evolutionary corollaries of socio-cultural influences on linguistic form. Lingua. 2007; 117: 543–578.

[8] Lashley KS. The problem of serial order in behavior In: Jeffress LA, editor. Cerebral mechanisms in behavior. New York: Wiley; 1951 pp. 112–146.

[9] Studdert-Kennedy M. Some developments in research on language behavior In: Smelser NJ, Gerstein DR, editors. Behavioral and social science: Fifty years of discovery: In commemoration of the fiftieth anniversary of the “Ogburn Report: Recent Social Trends in the United States”. Washington, DC: National Academy Press; 1986 pp. 208–248.

[10] Wilbur RB, Nolkn SB. The duration of syllables in American Sign Language. Lang Speech. 1986; 29: 263–280. 3695760 10.1177/002383098602900306

[11] Remez RE, Ferro DF, Dubowski KR, Meer J, Broder RS, Davids ML. Is desynchrony tolerance adaptable in the perceptual organization of speech? Atten Percept Psychophys. 2010; 72: 2054–2058. 10.3758/APP.72.8.2054 21097850

[12] Pashler H. Familiarity and visual change detection. Percept Psychophys. 1988; 44: 369–378. 3226885 10.3758/bf03210419

[13] Cowan N. The magical number 4 in short-term memory: A reconsideration of mental storage capacity. Behav Brain Sci. 2000; 24: 87–185. 10.1017/s0140525x01003922 11515286

[14] Miller G. The magical number seven, plus or minus two: Some limits on our capacity for processing information. Psych Rev. 1956; 63: 81–97. 13310704

[15] Wilson M, Emmorey K. Comparing sign language and speech reveals a universal limit on short-term memory capacity. Psychol Sci. 2006; 17: 682–683. 10.1111/j.1467-9280.2006.01766.x 16913950

[16] Christiansen MH, Chater N. The Now-or-Never bottleneck: A fundamental constraint on language. Behav Brain Sci. 2016; 39: e62 10.1017/S0140525X1500031X 25869618

[17] Ding N, Melloni L, Zhang H, Tian X, Poeppel D. Cortical tracking of hierarchical linguistic structures in connected speech. Nat Neurosci. 2016; 19: 158–164. 10.1038/nn.4186 26642090 PMC4809195

[18] Stephens GJ, Honey CJ, Hasson U. A place for time: the spatiotemporal structure of neural dynamics during natural audition. J Neurophysiol. 2013; 110: 2019–2026. 10.1152/jn.00268.2013 23926041 PMC3841928

[19] Arnon I, Clark EV. Why brush your teeth is better than teeth–Children's word production is facilitated in familiar sentence-frames. Lang Learn Dev. 2011: 107–129.

[20] Bannard C, Matthews D. Stored word sequences in language learning the effect of familiarity on children's repetition of four-word combinations. Psychol Sci. 2008; 19: 241–248. 10.1111/j.1467-9280.2008.02075.x 18315796

[21] Tomasello M, Constructing a language: A usage-based theory of language acquisition Cambridge, MA: Harvard University Press; 2003.

[22] Arnon I, Snider N. More than words: Frequency effects for multi-word phrases. J Mem Lang. 2010; 62: 67–82.

[23] Janssen N, Barber HA. Phrase frequency effects in language production. PLoS ONE. 2012; 7(3): e33202 10.1371/journal.pone.0033202 22479370 PMC3314013

[24] Reali F, Christiansen MH. Processing of relative clauses is made easier by frequency of occurrence. J Mem Lang. 2007; 57: 1–23.

[25] Scott-Phillips T, Kirby S. Language evolution in the laboratory. Trends Cogn Sci. 2010; 14: 411–417. 10.1016/j.tics.2010.06.006 20675183

[26] Kirby S, Cornish H, Smith K. Cumulative cultural evolution in the laboratory: An experimental approach to the origins of structure in human language. Proc Nat Acad Sci. 2008; 105: 10681–10685. 10.1073/pnas.0707835105 18667697 PMC2504810

[27] Tria F, Galantucci B, Loreto V. Naming a structured world: a cultural route to duality of patterning. PLOS one. 2012; 7(6): e37744 10.1371/journal.pone.0037744 22723839 PMC3378539

[28] Lee Y, Collier TC, Stabler EP, Taylor CE. The role of population structure in language evolution. Proceedings of the 10th International Symposium on Artificial Life and Robotics. Beppu, Oita, Japan. 2005.

[29] Perfors A, Navarro DJ. Language evolution can be shaped by the structure of the world. Cogn Sci. 2014; 38: 775–793. 10.1111/cogs.12102 24460933

[30] Chomsky N. Language and other cognitive systems. What is special about language? Lang Learn Dev. 2011; 7: 263–278.

[31] Galantucci B, Garrod S. Experimental semiotics: a review. Frontiers Human Neuroscience. 2011; 5:11. 10.3389/fnhum.2011.00011 PMC3043271 21369364

[32] Feher O, Wang H, Saar S, Mitra P, Tchernichovski O. De novo establishment of wild-type song culture in the zebra finch. Nature. 2009; 459: 564–568. 10.1038/nature07994 19412161 PMC2693086

[33] Claidière N, Smith K, Kirby S, Fagot J. (2014). Cultural evolution of systematically structured behaviour in a non-human primate. Proc R Soc B. 2014; 281:20141541 10.1098/rspb.2014.1541 25377450 PMC4240982

[34] Galantucci B. An experimental study of the emergence of human communication systems. Cogn Sci. 2006; 29: 737–767. 10.1207/s15516709cog0000_34 21702792

[35] Christiansen MH, Chater N. Language as shaped by the brain. Behav Brain Sci. 2008; 31: 489–509. 10.1017/S0140525X08004998 18826669

[36] Verhoef T, Kirby S, de Boer B. (2014). Emergence of combinatorial structure and economy through iterated learning. J Phonetics. 2014; 43C: 57–68.

[37] Smith K, Wonnacott E. Eliminating unpredictable variation through iterated learning. Cognition. 2010; 16: 444–449. 10.1016/j.cognition.2010.06.004 20615499

[38] Levenshtein VI. Binary codes capable of correcting deletions, insertions and reversals. Soviet Physics-Doklady. 1966; 10; 707–710.

[39] Vokey JR, Brooks LR. Salience of item knowledge in learning artificial grammars. J Exp Psychol Learn Mem Cogn. 1992; 18: 328–344.

[40] Barr DJ, Levy R, Scheepers C, Tily HJ. (2013). Random effects structure for confirmatory hypothesis testing: Keep it maximal. J Mem Lang. 2013; 68, 255–278. 10.1016/j.jml.2012.11.001 PMC3881361 24403724

[41] Knowlton BJ, Squire LR. The information acquired during artificial grammar learning. J Exp Psych: Learn Mem Cogn. 1994; 20: 79–91. 10.1037//0278-7393.20.1.79 8138790

[42] MacWhinney B. The CHILDES Project: Tools for Analyzing Talk. Mahwah, NJ: Lawrence Erlbaum Associates; 2000.

[43] Baronchelli A, Ferrer-i-Cancho R, Pastor-Satorras R, Chater N, Christiansen MH. Networks in cognitive science. Trends Cogn Sci. 2013; 17: 348–360. 10.1016/j.tics.2013.04.010 23726319

[44] Chan KY, Vitevitch MS. Network structure influences speech production. Cogn Sci. 2010; 34: 685–697. 10.1111/j.1551-6709.2010.01100.x 21564230

[45] Ferrer-i-Cancho R, Solé RV, Köhler R. (2004). Patterns in syntactic dependency networks. Physical Rev E. 2004; 69: 051915. 10.1103/PhysRevE.69.051915 15244855

[46] Sindi SS, Dale R. Culturomics as a data playground for tests of selection: Mathematical approaches to detecting selection in word use. J Theor Biol. 2016; 405: 140–149. 10.1016/j.jtbi.2015.12.012 26802483

[47] Towse JN, Towse AS, Saito S, Maehara Y, Miyake A. Joint cognition: thought contagion and the consequences of cooperation when sharing the task of random sequence generation. PLoS ONE. 2016; 11(3): e0151306 10.1371/journal.pone.0151306 26977923 PMC4792471

[48] Ginsburg N, Karpiuk P. Random generation: Analysis of the responses. Percept Motor Skills. 1994; 79: 1059–1067.

[49] Winter B, Matlock T. More is up… and right: Random number generation along two axes. Proceedings of the 35th annual conference of the Cognitive Science Society, 2013: 3789–3974. Austin, TX: Cognitive Science Society.

[50] Towse JN, Neil D. Analyzing human random generation behavior: A review of methods used and a computer program for describing performance. Behav Res Meth Ins C. 1998; 30: 583–591.

[51] Kamada T, Kawai S. An algorithm for drawing general undirected graphs. Inf Proc Lets. 1989; 31: 7–15.

[52] Kirby S, Hurford J. The emergence of linguistic structure: an overview of the iterated learning model In Cangelosi A, Parisi D, editors. Simulating the evolution of language. London: Springer-Verlag; 2002; pp. 121–147.

[53] Christiansen MH, Dale RA, Ellefson MR, Conway CM. The role of sequential learning in language evolution: Computational and experimental studies In Cangelosi A, Parisi D, editors. Simulating the evolution of language. London: Springer-Verlag; 2002; pp. 165–187.

[54] Zipf G. Human behavior and the principle of least effort. New York: Addison-Wesley 1949.

[55] Croft W. Syntactic categories and grammatical relations: The cognitive organization of information. Chicago: University of Chicago Press 1991.

[56] Hawkins JA. A performance theory of order and constituency. Cambridge, UK: Cambridge University Press 1994.

[57] Kirby S. Function, selection and innateness: the emergence of language universals Oxford: Oxford University Press 1999.

[58] Kemp C, Regier T. Kinship categories across languages reflect general communicative principles. Science. 2012; 336: 1049–1054. 10.1126/science.1218811 22628658

[59] Garrod S, Fay N, Walker B, Swoboda N. Can iterative learning explain the emergence of graphical symbols? Interact Stud. 2010; 11: 33–50.

[60] Smith K, Kirby S. Cultural evolution: implications for understanding the human language faculty and its evolution. Phil Trans R Soc B. 2008; 363: 3591–3603. 10.1098/rstb.2008.0145 18801718 PMC2607345

[61] Blevins J, Wedel A. Inhibited sound change: An evolutionary approach to lexical competition. Diachronica. 2009; 26: 143–183.

[62] Wedel AB. Lexical contrast maintenance and the organization of sublexical contrast systems. Lang Cogn. 2012; 4: 319–356.

[63] Gobet F, Lane PC, Croker S, Cheng PC, Jones G, Oliver I, Pine JM. Chunking mechanisms in human learning. Trends Cogn Sci. 2001; 5: 236–243. 11390294 10.1016/s1364-6613(00)01662-4

[64] Jones G, Gobet F, Freudenthal D, Watson SE, Pine JM. Why computational models are better than verbal theories: the case of nonword repetition. Dev Sci. 2014; 17: 298–310. 10.1111/desc.12111 24238080

[65] Christiansen MH, Chater N. Creating language: Integrating evolution, acquisition, and processing Cambridge, MA: MIT Press; 2016.

