# Supplementary material for: Sequence Memory Constraints Give Rise to Language-Like Structure through Iterated Learning
Source: PLoS One. 2017 Jan 24;12(1):e0168532. doi: 10.1371/journal.pone.0168532 (PMC5261806; doi:10.1371/journal.pone.0168532)
Supplement: S1 Text — (DOCX) [file pone.0168532.s001.docx]

**Supporting Material: String Length-Connectivity Scaling**

The scaling of string connectivity with string length, shown in Fig 3 in the main text, may be a common property of naturally sequenced strings across many domains. For this reason, it may not be reflective of *psychological* properties that would form the basis of the patterns of reuse we are proposing for language. We tested three other string sets to demonstrate that this scaling is in fact not a property of all natural human-generated sequences.

*Google Ngram Statistics*

We used a subset of English words found in Google Ngram (books.google.com/ngrams) from a prior study [46]. This subset included only highly frequent words across the century from 1900 to 2000. We treated the frequencies of these words as strings. The numbers that make up these frequencies would form the strings of various length comparable to those we used in Fig 3 from the human experiments. Because this closed set was of highly frequent words, string lengths varied from 4 to 7. For example, the word “sadly” had a frequency of 7,073 occurrences in 1963. “7073” was thus used as a string of length four, and we connected it to other frequency counts with which it shares bigrams (two-digit numbers). The word “sad,” in 1965, has a frequency of 25,084 (string of length 5). Because these numbers do not share a two-digit number sequence, they are not connected in the overall network. These frequencies are not human generated in and of themselves but, rather, are a reflection of the overall frequency of usage across many humans. The particular number should not show reuse in the manner that we demonstrated in experimental and CHILDES networks.

We sampled 5,000 word frequencies across the century of 1900 to 2000, and used these as our string set. When building Fig 4 (left panel) for this random set of 5,000 such words, we see that bigram connectivity does not scale differently for shuffled and original data. Using the same regression approach with two predictors (length and shuffled/unshuffled), there is only a main effect of length present in this sample of data (p < .0001), while length and shuffling do not interact (p = .14).

*Numbers Occurring in Passwords*

A human-generated sequence that may show reuse pattern are passwords. Such passwords are individually produced by each person, and the numbers employed in passwords (e.g., “JustinBieberFan1942” => “1942”) can be extracted from a database of passwords online (10 Million Passwords: xato.net/today-i-am-releasing-ten-million-passwords-b6278bbe7495). We only analyzed the numbers in password because, of course, the remainder of any password may be composed of words or phrases and thus would bias towards a language-like scaling that we observe in the CHILDES data. A randomly selected set of 5,000 such passwords was sampled, and numbers extracted. This produces approximately 1,000 numbers, and suggests a slight benefit of the unshuffled data, showing slightly more connectivity, but it is not so pronounced as the reuse data in our human experiments (Fig 4, center panel). Again, a regression model with length and shuffled/unshuffled produces no significant interaction (p = .34), and only a main effect of length (p < .0001). There is a positive effect of preserving the original data (p = .02), in that observed digits tend to have more shared structure overall, but the lack of interaction suggests this is not a function of string length, as expected by the human experimental and CHILDES data.

*Random Number Generation*

If humans indeed rely on reuse during sequential processing to facilitate memory, then when one person generates a sequence of digits for an extended time, we may see patterns of reuse that might resemble the scaling we observed in Fig 3. To test this, we obtained data from a random number generation task [47]. Participants randomly generated numbers from 1 to 10 for 100 trials. This produces one long string of digits. We randomly sampled substrings of this longer digit sequence, from lengths 3 to 6. This produces a new set of strings of varying length representing windows of digits that were randomly generated by the participants in this study. Taking 5 of these subjects for our analysis, we end up with about 100 such digit sequences. Scanning for shared bigrams, we can once more test for connectivity across these substrings. Again, as indicated by Fig 4 (right panel), there appears to be no apparent scaling, and neither main effect (p = .9) nor interaction is significant (p = .5).
